# Supplementary material for: Revealing the Innate Subnanometer Porous Structure of Carbon Nanomembranes with Molecular Dynamics Simulations and Highly-Charged Ion Spectroscopy
Source: J Phys Chem C Nanomater Interfaces. 2026 Mar 10;130(11):4244–55. doi: 10.1021/acs.jpcc.5c08242 (PMC13007017; doi:10.1021/acs.jpcc.5c08242)
Supplement: Supplementary file 1 [file jp5c08242_si_001.pdf]

Revealing the Innate Sub-Nanometer Porous Structure of Carbon Nanomembranes with Molecular Dynamics Simulations and Highly-Charged Ion Spectroscopy: Supplementary Information

Filip Vuković,[*,†,⊥] Anna Niggas,[†,⊥] Levin Mihlan,[‡] Zhen Yao,[‡] Armin Gölzhäuser,[‡] Louise Fréville,[¶] Vladislav Stroganov,[§] Andrey Turchanin,[§] Jürgen Schnack,[‡] Nigel A. Marks,[‖] and Richard A. Wilhelm[†]

†*Institute of Applied Physics, TU Wien, Vienna, 1040, Austria*

‡*Faculty of Physics, Bielefeld University, Bielefeld, 33615, Germany*

¶*Phelma INP Grenbole, Grenoble, 38000, France*

§*Institute of Physical Chemistry, Friedrich Schiller University Jena, Jena, 07743, Germany*

‖*Department of Physics, Curtin University, Perth, 6102, Australia*

⊥*Contributed equally to this work*

E-mail: vukovic@iap.tuwien.ac.at

Experimental spectra

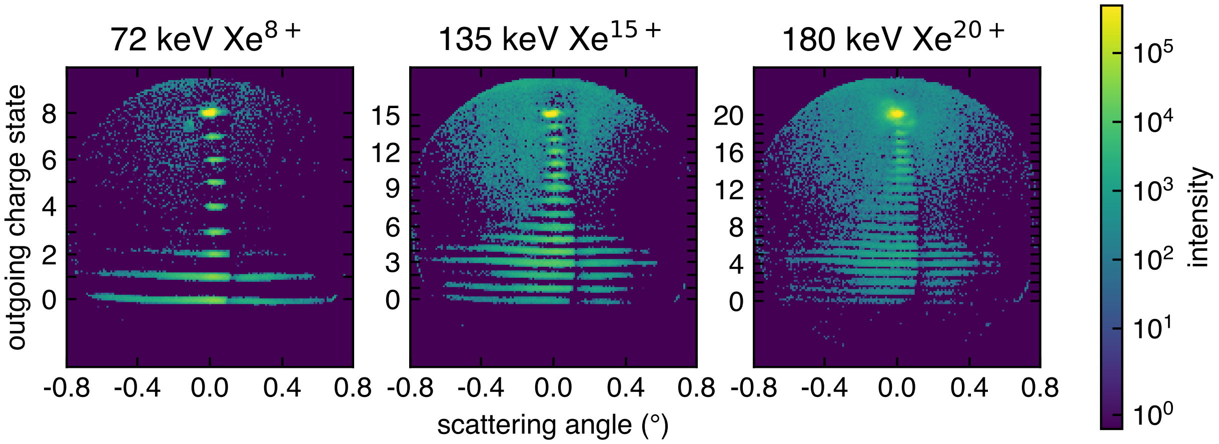

Figure S1: Experimental 2D charge exchange spectra colormap for xenon on TPT carbon nanomembrane with background noise removed. Here, the colour scale is logarithmic and maximal intensity is normalised to unity for each graph

Time-dependent potential simulations: tuning interatomic Coulomb decay for 3D targets

The Time Dependent Potential (TDPot) method was initially developed to model the transmission of highly charged ions on 2D materials, namely graphene. While this model was able to accurately predict ion-target charge exchange for Xe $\rightarrow$ single-layer graphene (SLG), it could not predict charge exchange for bi-layer and tri-layer graphene (BLG and TLG, respectively). Here, we modified the calculation of the γ function that mediates the interatomic Coulomb decay (ICD) of the ion such that it was suitable for targets with finite thickness. To this end, we performed a large set of benchmark simulations for a range of a, b, c parameters. Unit cells of single, bi-, and tri-layer graphene were used for the Xe ion transmission simulations, consisting of 60, 120 and 180 carbon atoms, respectively. For these simulations, as well as the production carbon nanomembrane (CNM) simulations, the Kr-C scattering potential[1] was used.

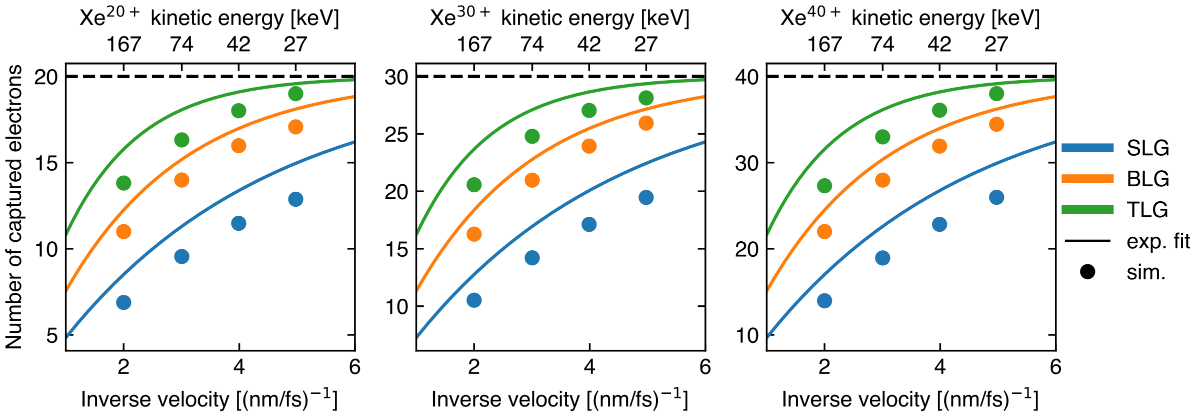

Figure S2: Simulated mean exit low-charge state from the charge exchange spectra of Xe ions incident on single-layer graphene (blue), bi-layer graphene (orange), and tri-layer graphene (green) shown in solid triangles for an incident charge state of 20 (panel a), 30 (panel b), and 40 (panel c), as a function of inverse ion velocity and compared to experimental fits from Ref. 2.

Figure S2 presents the simulated mean low exit charge state from the charge exchange distribution (integrated over all scattering angles) in solid triangles for Xe^{20+}, Xe^{30+}, Xe^{40+} (panels a), b) and c), respectively) incident on SLG (blue), BLG (orange), and TLG (green), as a function of initial inverse velocity, and compared to experimental fits from Ref. 2. Only the results from the best fitting gamma parameters is shown. In general, a good fit to the experimental data can be observed, with the TDPot simulations systematically underestimating the mean exit charge state for TLG. Table S1 summarises the gamma parameters used for the TDPot simulations reported in the main text.

Table S1: Gamma parameters used in the time-dependent potential calculations, noting that all units are in atomic units.

parameter	value [a.u.]
a	0.0105
b	2.8
c	2.0

Additional simulation results: enforced cylinder simulations

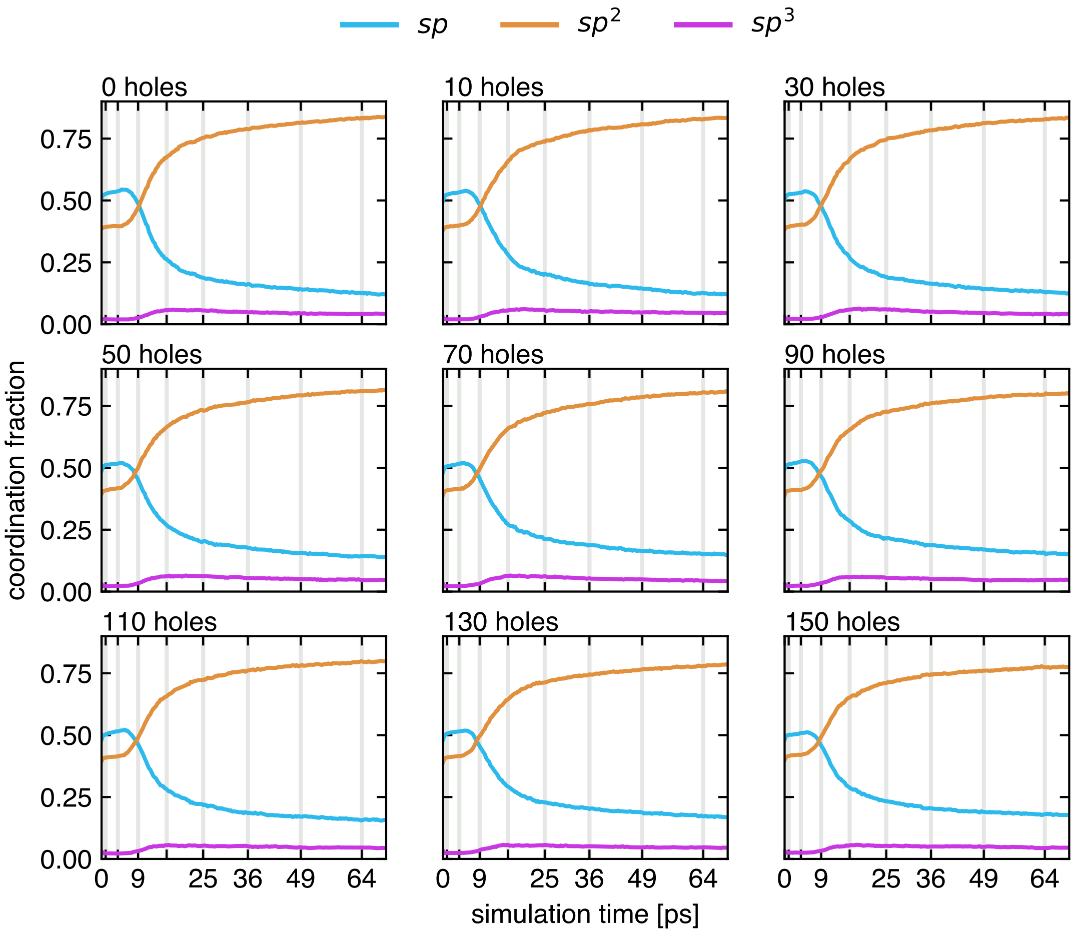

Figure S3: Coordination percentages as a function of simulation time, where blue lines indicate *sp* carbon, orange is sp^2, and magenta is sp^3. Light grey vertical lines indicate when trajectories were branched off to be quenched and produce final membrane structures.

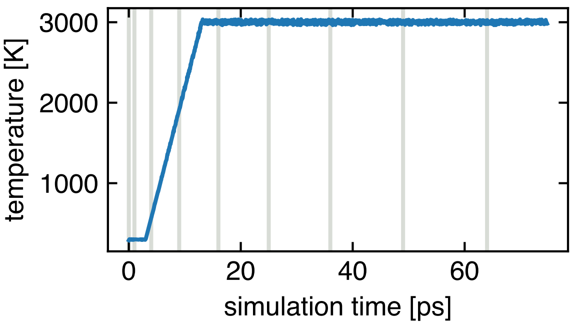

Figure S4: Example system temperature of the 0 exclusion cylinder simulation, where grey lines indicate timesteps from which new simulations were started from.

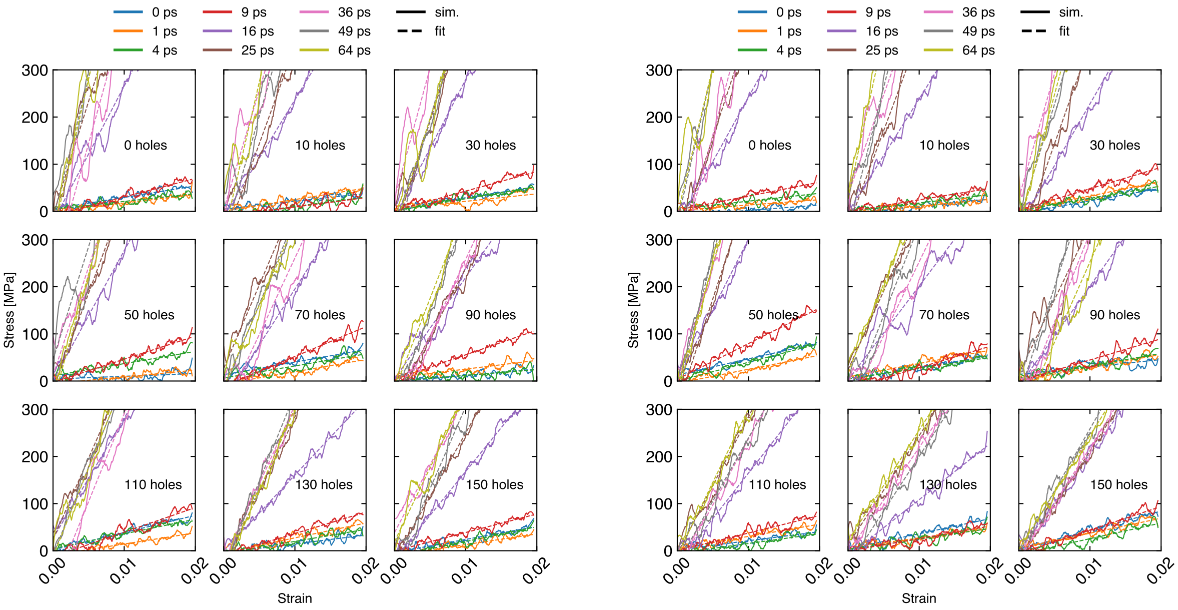

(a) (b)

Figure S5: Simulated stress-strain curves for the hole-enforced carbon nanomembrane (solid lines), with stress applied in the x (panel a) and the y (panel b) unit-cell directions. The dashed lines indicate the linear fits used to calculate the tensile moduli.

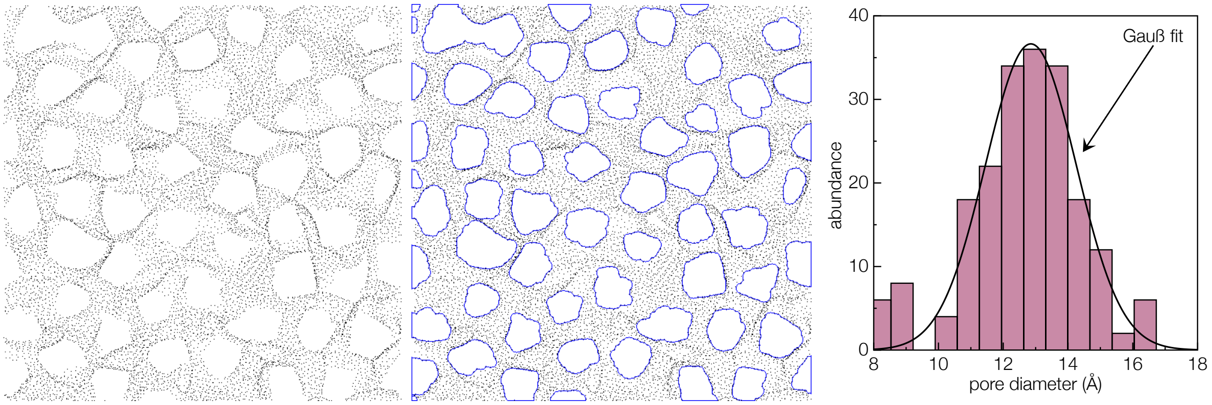

Figure S6: The pore analysis pipeline. Carbon atom positions of the maximally annealed 51-holes CNM structure (left panel), with the pore edge contours overlaid. (center panel). The resultant pore diameter histogram is shown on the right.

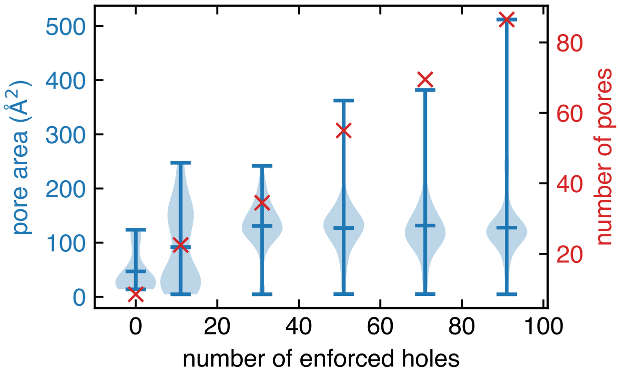

Figure S7: Pore area distributions (blue shaded regions) plotted as a function of hole-count for the maximally annealed CNM structures produced with the hole-enforced simulation method, with the distribution means and ranges indicated by horizontal blue lines. Red crosses indicate the number of detected pores with the y-scale shown on the right of the graph in red.

Additional simulation results: momentum-transfer simulations

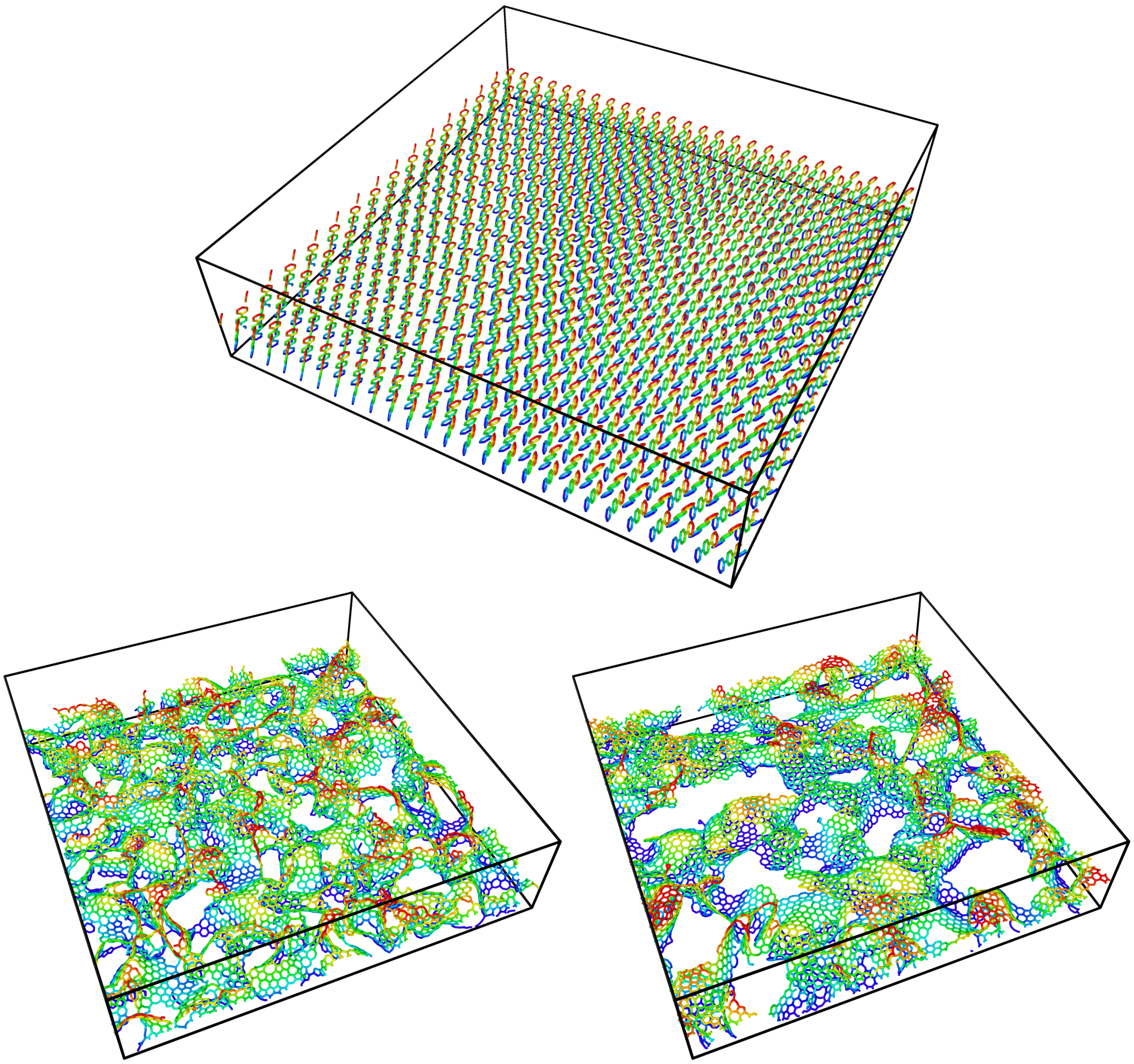

Figure S8: Top: Initial unit cell for the momentum transfer simulations: a self-assembled monolayer of the carbon atoms of the terphenyl thiol molecule, with other non-carbon elements removed. The unit cell was ≈ 135 Å x 134 Å x 40 Å, and the color indicates the distance from the substrate in z-directions (ranging from 0 Å (blue) to 10.7 Å (red)) containing 14040 carbon atoms in total. The unit cell boundary is indicated by the solid black lines. Bottom: Exemplary results from the momentum transfer simulation with attractive (left) and repulsive (right) events.

Time evolution of sp-fractions in formation process with attractive or repulsive events

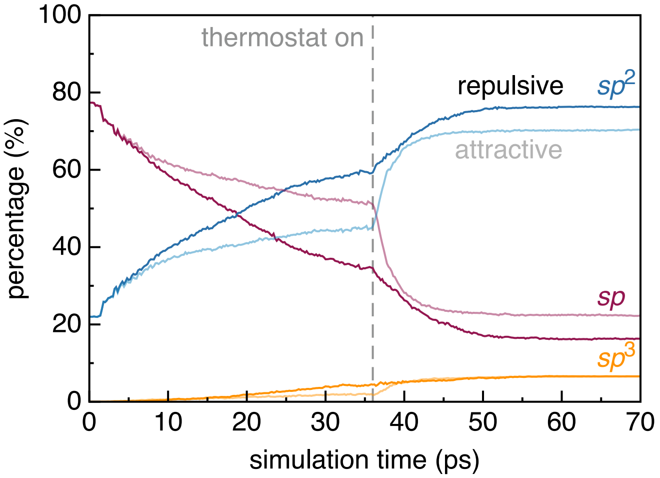

Figure S9: Carbon atom coordination percentages as a function of simulation time. Dashed line indicates time at which thermostat is activated. Attractive and repulsive events are depicted.

Test simulation were performed that explored the effect of the secondary force direction, i.e. the difference between a secondary force directed towards the primary force event site, and a secondary force directed away from the primary site, here in referred to as attractive, and repulsive, respectively. Figure S9 clearly shows that the sp^2 percentage at the end of the dynamic is higher in simulation with repulsive events than in the one with attractive events. The opposite behavior can be observed for the sp component. The sp^3 component is almost identical in both simulations.

Another striking feature is the rate of change in the shares over time. For example, while the sp^2 proportions increase at a similarly strong rate at the beginning, this rate decreases rapidly in the simulation with attractive events, while the rate of the repulsive event simulation remains almost constant with only a small decrease.

Yet another characteristic point is the time at which the sp^2 component exceeds the sp component. While this is already the case after around 90 frames with repulsive events, the intersection point in the simulation with attractive events does not occur until frame 190,

after the thermostat has already been activated. This indicates poorer crosslinking due to inwardly directed secondary forces, which results in the thermostat doing a lot of crosslinking work instead of just cooling the already crosslinked structure. This can be seen in the very sharp increase in the rate when the thermostat is switched on, in case of the graphic with attractive events.

According to this partial study, the simulation variant with outwardly directed forces has a higher crosslinking capability while using the same simulation parameters. Note that all production simulations discussing in the main manuscript used a repulsive secondary force.

Additional results

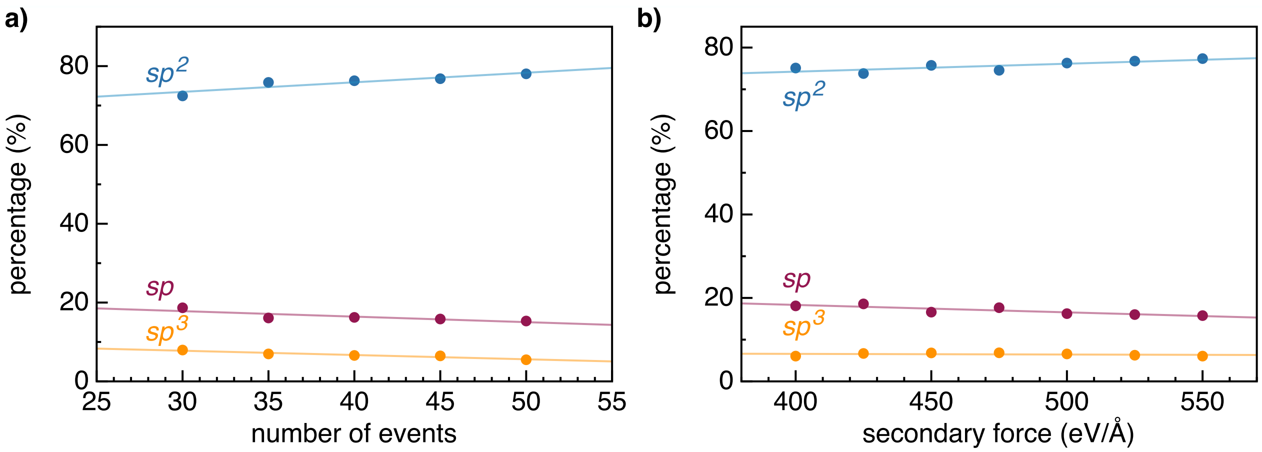

Figure S10: Carbon atom coordination percentages for the (a) event-varied simulations and (b) force-varied simulations. All other parameters were kept constant (time steps per event = 400; primary force = 50 eV/Å; fluctuations 300 K; evolving time steps = 2000; Langevin cooling = 350000). For (a) 40 events, and for (b) a secondary force of 500 eV/Å was used.

Additional experimental information

Sample preparation at the University of Bielefeld

Terphenyl thiol (TPT) CNMs were prepared following the protocol described in Ref 3 and nitro terphenyl thiol (NBPT) CNMs were prepared according to the method described in Ref 4.

In both cases, the Au/mica substrates (from Georg Albert PVD Deposition, Germany) were cleaned using a UV/ozone cleaner (UVOH 150 LAB FHR) for 3 minutes, immersed in absolute ethanol (≥99.8%, VWR Chemicals) for 20 minutes, and dried using nitrogen. The cleaned gold substrates were then immersed in 1 mmol solution of 1, 1', 4', 1'-terphenyl-4-thiol (TPT, Sigma-Aldrich) and ∼10 mmol solution of 4'-nitro 1,1'-biphenyl-4-thiol (NBPT, Taros Chemicals) in dry and degassed dimethylformamide (anhydrous, 99.8%, Sigma-Aldrich) at 70°C under an inert atmosphere. The incubation time was 24 hours for TPT and 72 hours for NBPT.

Following self-assembly, the samples were rinsed with DMF and ethanol, dried with nitrogen, and converted into CNMs via 100 eV electron exposure with a dose of 50 mC cm^{-2} in a high vacuum chamber ($< 8 \times 10^{-8}$ mbar). To stabilize the CNMs during transfer, they were coated with polymethyl methacrylate (PMMA, AR-P 671.04) in ethyl acetate (Fisher Chemical). The PMMA/CNM/Au stack was delaminated from the mica support by dipping into water, and the Au layer was etched using an I2/KI/H2O solution (1:4:10; iodine, 99.8%, Alfa Aesar; potassium iodide, ≥99%, Carl Roth). Finally, the PMMA/CNM stack was transferred onto a TEM-grid, baked at 90°C for 1 hour, and the PMMA coating was removed by immersion in acetone (≥98%, Fisher Chemical) for 1 hour.

XPS measurements were performed for all samples and results are summarised in Figure S11.

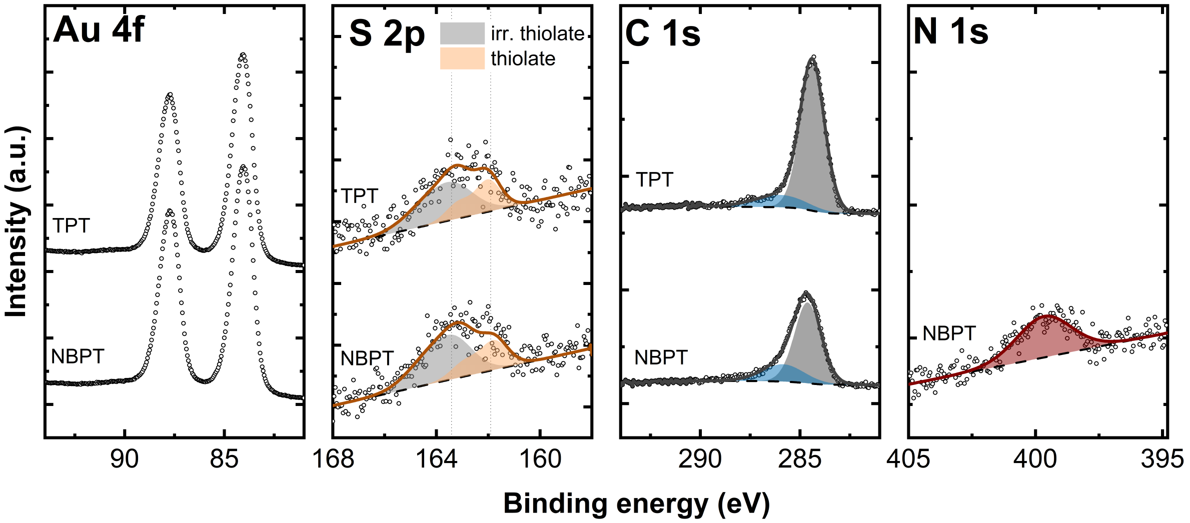

Figure S11: XPS measurements of the carbon nano membranes prepared at the University of Bielefeld.

Cleaning nitro biphenyl thiol CNMs

Other than in experiments performed ten years ago, no upper charge state distribution was visible for nitro biphenyl thiol CNMs in their pristine form. Hence, the cleaning procedure described in the main manuscript was also applied to these membranes. Analogue to the terphenyl thiol data presented in Figure 1 in the main manuscript, Figure S12 shows the mean exit charge state after transmission through an nitro biphenyl thiol CNM as a function of the applied fluence for the lower charge states (neutral Xe to Xe^{4+} in blue), and and the higher charge states (Xe^{5+} to Xe^{9+} in red). The low charge state distribution plateaus similar to the terphenyl thiol case after an applied fluence on the order of 10^{11} ions/cm^2.

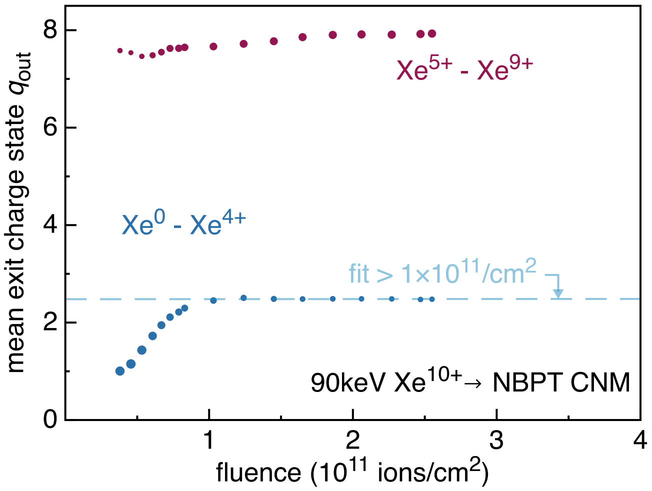

Figure S12: Mean exit charge states of the low (blue) and high (red) exit charge state distribution of 90 keV Xe^{10+} transmitted through an nitro biphenyl thiol carbon nanomembrane (CNM). A fluence of $\sim 1 \times 10^{11}$ ion impacts per cm^2 is necessary to reach an equilibrium charge state of $q_{\text{out}} \sim 2$.

Comparison of suppliers and precursor molecules

The neutralisation behaviour of highly charged ions of different charge states q_{in} transmitted through CNMs with nitro biphenyl thiol and terphenyl thiol precursors is compared in Figure S13 (a). All measurements were performed with the same acceleration potential, i.e., the kinetic energy for each charge state amounts to $E_{\text{kin}} = 9.6 \times q_{\text{in}}$ keV. Both exit charge state distributions (high charge states q_{high} and low charge states q_{low}) are lower for terphenyl thiol membranes compared to NBPT membranes. For q_{high} this probably is a direct consequence of the different material thicknesses.

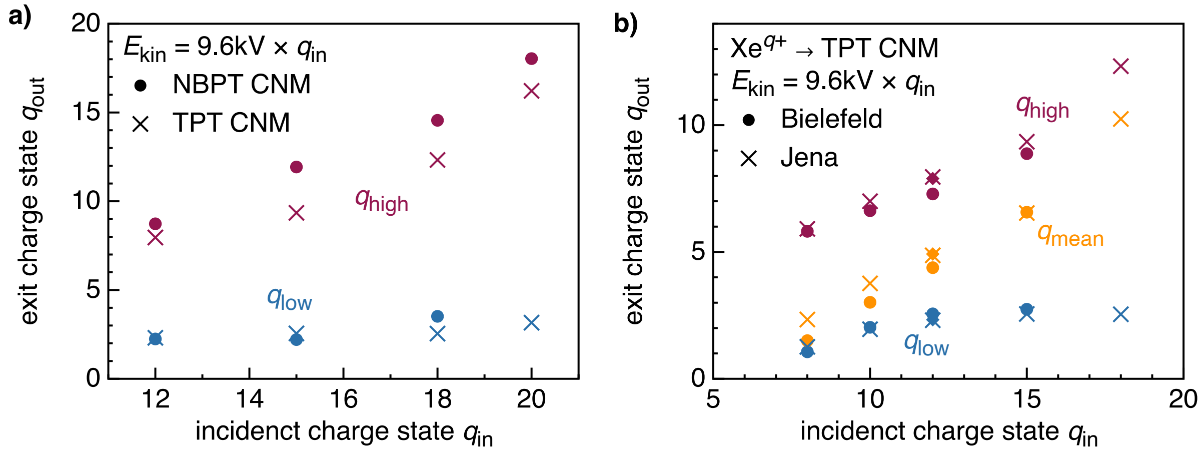

Figure S13: Comparison of (a) carbon nanomembranes (CNMs) with nitro biphenyl thiol (NBPT) (dots) and terphenyl thiol (TPT) (crosses) precursors prepared at the University of Jena and (b) terphenyl thiol CNMs from the University of Jena (crosses) and Bielefeld (dots). Mean exit charge states of the high (q_{high}, red) and low (q_{low}, blue) distributions are shown. In (b) the overall mean exit charge state (q_{mean}, orange) is also added.

A comparison of different sample preparation procedures, in this case at the Universities of Jena and Bielefeld, respectively, is shown in Figure S13. Again, different incident charge states were used for transmission experiments with constant acceleration potential. Good agreement is observed for the overall mean exit charge state (q_{mean}, orange) as well a q_{high} and q_{low}.

References

(1) Wilson, W. D.; Haggmark, L. G.; Biersack, J. P. Calculations of nuclear stopping, ranges, and straggling in the low-energy region. *Phys. Rev. B* **1977**, *15*, 2458–2468.

(2) Niggas, A.; Creutzburg, S.; Schwestka, J.; Wöckinger, B.; Gupta, T.; Grande, P. L.; Eder, D.; Marques, J. P.; Bayer, B. C.; Aumayr, F.; Bennett, R.; Wilhelm, R. A. Peeling graphite layer by layer reveals the charge exchange dynamics of ions inside a solid. *Commun. Phys.* **2021**, *4*.

(3) Angelova, P.; Vieker, H.; Weber, N.-E.; Matei, D.; Reimer, O.; Meier, I.; Kurasch, S.; Biskupek, J.; Lorbach, D.; Wunderlich, K.; others A universal scheme to convert aromatic molecular monolayers into functional carbon nanomembranes. *ACS nano* **2013**, *7*, 6489–6497.

(4) Eck, W.; Stadler, V.; Geyer, W.; Zharnikov, M.; Gölzhäuser, A.; Grunze, M. Generation of Surface Amino Groups on Aromatic Self-Assembled Monolayers by Low Energy Electron Beams—A First Step Towards Chemical Lithography. *Advanced Materials* **2000**, *12*, 805–808.
